# Hyoscine in Paralysis Agitans

**Published:** 1902-05-03

**Authors:** 


[1] Lancet, April 12.

### HYOSCINE IN PARALYSIS AGITANS.

Dr. Judson S. Bury[1] says that hyoscine is probably the most useful drug that has hitherto been tried in the treatment of paralysis agitans. As a rule, it diminishes or arrests the tremor, checks the troublesome restlessness and the desire to change position, and relieves the "hot flushes" and unpleasant sensations of heat by which these patients are often troubled. It is important to remember, however, that hyoscine is a powerful drug and one which must be administered with great care. Merck's hyoscine hydrobromate is probably the best preparation, and it is safer to give it by the mouth than to inject it. Quoting from Williamson, he says, "A prescription which is useful is one-eighth of a grain of hyoscine hydrobromate in six ounces of chloroform water. At first two teaspoonfuls of this may be given, then three, four, or five teaspoonfuls. If necessary, the dose may be increased to six teaspoonfuls ($\frac{1}{64}$th of a grain), providing toxic symptoms are not produced. The hyoscine is best given in the morning, just after breakfast, and again in the evening, just before going to bed, if the patient is troubled with restlessness and sleeplessness during the night."

